# Terlipressin versus Norepinephrine in the Treatment of Hepatorenal Syndrome: A Systematic Review and Meta-Analysis

**DOI:** 10.1371/journal.pone.0107466

**Published:** 2014-09-09

**Authors:** Antonio Paulo Nassar Junior, Alberto Queiroz Farias, Luiz Augusto Carneiro d’ Albuquerque, Flair José Carrilho, Luiz Marcelo Sá Malbouisson

**Affiliations:** 1 Intensive Care Unit, Department of Gastroenterology, University of Sao Paulo. Sao Paulo, SP, Brazil; 2 Discipline of Gastroenterology, Department of Gastroenterology. University of Sao Paulo. Sao Paulo, SP, Brazil; 3 Liver and Gastrointestinal Transplant Division, Department of Gastroenterology. University of São Paulo, São Paulo, SP, Brazil; University Hospital Heidelberg, Germany

## Abstract

**Background:**

Hepatorenal syndrome (HRS) is a severe and progressive functional renal failure occurring in patients with cirrhosis and ascites. Terlipressin is recognized as an effective treatment of HRS, but it is expensive and not widely available. Norepinephrine could be an effective alternative. This systematic review and meta-analysis aimed to evaluate the efficacy and safety of norepinephrine compared to terlipressin in the management of HRS.

**Methods:**

We searched the Medline, Embase, Scopus, CENTRAL, Lilacs and Scielo databases for randomized trials of norepinephrine and terlipressin in the treatment of HRS up to January 2014. Two reviewers collected data and assessed the outcomes and risk of bias. The primary outcome was the reversal of HRS. Secondary outcomes were mortality, recurrence of HRS and adverse events.

**Results:**

Four studies comprising 154 patients were included. All trials were considered to be at overall high risk of bias. There was no difference in the reversal of HRS (RR = 0.97, 95% CI = 0.76 to 1.23), mortality at 30 days (RR = 0.89, 95% CI = 0.68 to 1.17) and recurrence of HRS (RR = 0.72; 95% CI = 0.36 to 1.45) between norepinephrine and terlipressin. Adverse events were less common with norepinephrine (RR = 0.36, 95% CI = 0.15 to 0.83).

**Conclusions:**

Norepinephrine seems to be an attractive alternative to terlipressin in the treatment of HRS and is associated with less adverse events. However, these findings are based on data extracted from only four small studies.

## Introduction

Hepatorenal syndrome (HRS) is a severe functional renal failure occurring in patients with cirrhosis and ascites. It develops as a consequence of the severe reduction in the renal perfusion secondary to splanchnic arterial vasodilation. Arterial vasodilation leads to a decrease in the effective blood volume, homeostatic activation of vasoactive systems (renin-angiotensin-aldosterone system [RAAS], antidiuretic hormone [ADH] and sympathetic nervous system) and, consequently, renal vasoconstriction [1].

HRS is sub-classified into types 1 and 2. Type 1 HRS is characterized by rapid progressive renal failure, usually accompanied by multiorgan failure. Type 2 HRS manifests itself as a slowly progressive functional renal failure associated with refractory ascites [1]. A 40% premature mortality rate has been reported in type 1 HRS [2], but may be as high as 83% [3]. Mortality associated with type 2 HRS ranges from 20% to 60% [2], [3]. Since the arterial vasodilation seems to be a key mechanism in the pathogenesis of HRS, vasoconstrictors have been used as a bridging therapy leading up to the definitive treatment; liver transplantation. The vasopressin analog terlipressin is the most widely studied drug, especially in type 1 HRS [4]. However, it is expensive and unavailable in many countries. Norepinephrine, a catecholamine with predominantly alpha-adrenergic activity, is widely available, inexpensive and has been used for the treatment of HRS type 1 since 2002 [5].

With the ominous prognosis of HRS and the high cost associated with terlipressin in mind, we performed a systematic review and meta-analysis to evaluate the efficacy and safety of norepinephrine compared to terlipressin in the treatment of HRS.

## Methods

### Literature Search

Studies were identified through a search of the Medline, EMBASE, Scopus, Cochrane Central Register of Controlled Trials (CENTRAL), Lilacs (*Literatura Latino-Americana e do Caribe em Ciências da Saúde*) and Scielo (*Scientific Eletronic Library Online*) databases. A sensitive search strategy was used, combining the following Medical Subject Headings and keywords: “terlipressin” and “norepinephrine” or “noradrenalin” in combination with “hepatorenal syndrome”. References of the included studies were also searched. The search strategy was restricted to randomized clinical trials performed on adult subjects and published before 14 January 2014. There was no language restriction. Titles and abstracts were assessed for eligibility and full-text copies of all articles deemed to be potentially relevant were retrieved. A standardized eligibility assessment was performed independently by two reviewers (APNJ and LMSM). Disagreements were resolved by consensus.

The PRISMA statement was used for guidance [6] and the meta-analysis was registered on the PROSPERO database (CRD42013006723).

### Study selection

Studies that fulfilled the following criteria were included:

1. Compared terlipressin to norepinephrine in the treatment of type 1 or type 2 HRS;
2. Reported at least one of the following outcomes: reversal of HRS, effect on mortality, recurrence rates after cessation of the treatment or assessment of adverse events on both arms of the study.

### Data extraction and quality assessment

A data extraction sheet was developed. Two authors (APNJ and LMSM) independently extracted the following data from included studies, as available: year of publication, number of patients designated to terlipressin or norepinephrine, methods of randomization, allocation concealment, blinding method, age, type of HRS, etiology of cirrhosis and duration of treatment. Child-Pugh and MELD scores, serum creatinine and mean arterial pressure (MAP) were recorded at baseline. Authors of the included studies were contacted by email to complete the missing data that was required for characterizing the studies.

Two authors (APNJ and LMSM) assessed the risk of bias of individual trials using the Cochrane risk of bias tool [7]. For the outcomes in each included trial, the risk of bias was reported as ‘low risk’, ‘unclear risk’, or ‘high risk’ in the following domains: random sequence generation; allocation concealment; blinding of participants and personnel; blinding of outcome assessment; incomplete outcome data; selective reporting; or other bias. Disagreements were resolved by consensus.

### Outcome measurements

The primary outcome was the reversal of HRS, defined as a decrease in the serum creatinine value to 133 µmol/l (1.5 mg/dl) or lower during the treatment. Secondary outcomes were mortality, recurrence of HRS and adverse effects.

### Statistical Analysis

Heterogeneity was assessed by the I^2^ statistic. A random-effects model was employed due to the anticipated variability between trials in terms of patient populations, interventions, and concomitant interventions. The effect of the treatment on the defined outcome measures was calculated from the raw data using random effects models. Differences observed between the treatment groups were expressed as the pooled risk ratio (RR) with a 95% confidence interval (CI). *A priori* subgroup analysis was performed to assess reversal, mortality and recurrence of type 1 and type 2 HRS. All analyses were performed using STATA version 13.0 (STATA Corporation, College Station, TX, USA) and Open Meta Analyst [8].

## Results

### Trial identification

**Figure 1:**
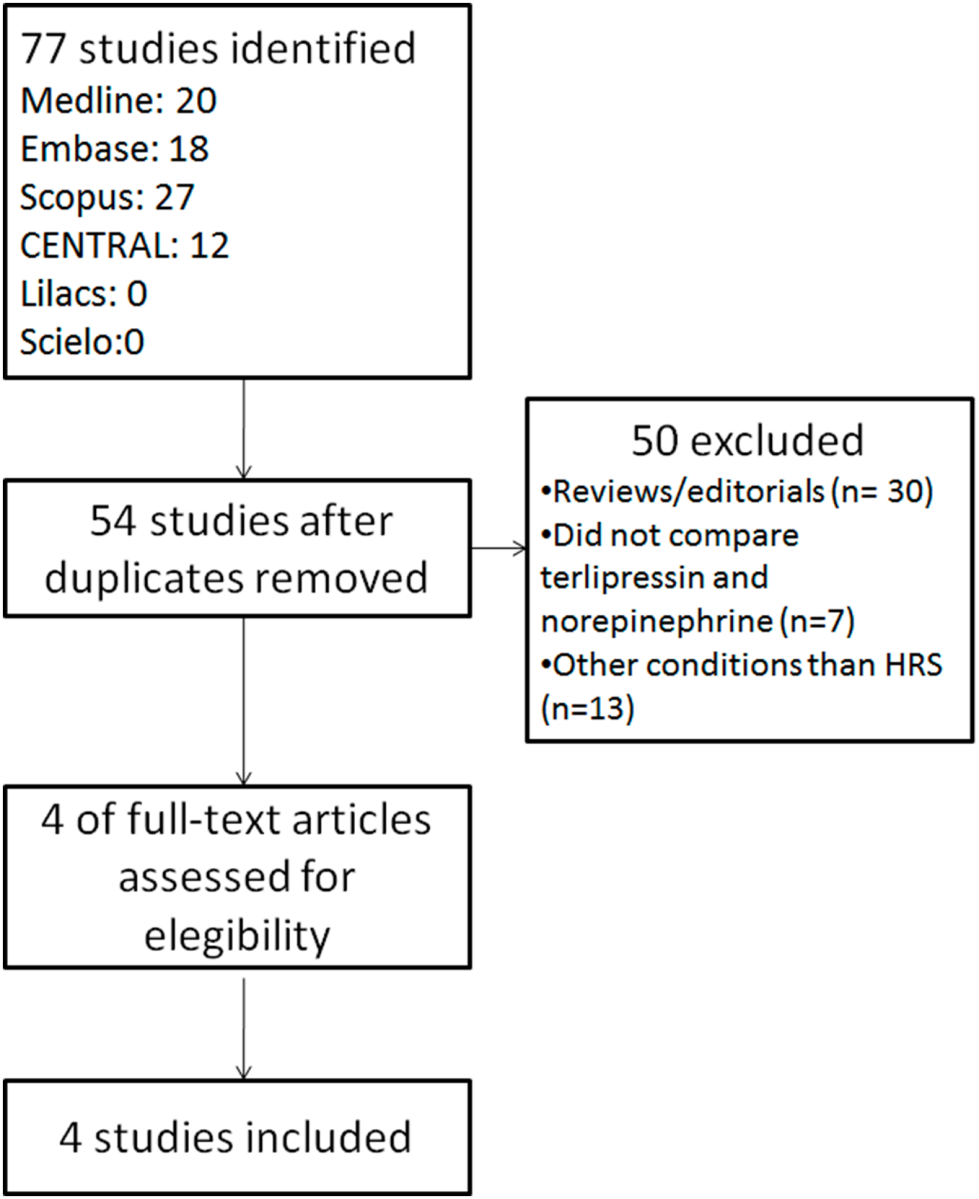
Search strategy.

The search yielded 77 publications. Four randomized controlled trials were selected for the analysis (Figure 1) [9], [10], [11], [12].

### Trial characteristics

Table 1 summarizes the details of included studies. One study was performed in Italy [9] and the remaining three were performed at the same center in India [10], [11], [12]. Two studies included patients with type 1 HRS [10], [11], one with type 2 HRS [12] and one with both types of HRS [9]. The studies performed by Singh et al. [11] and Ghosh et al. [12] were actually a single center trial which randomized patients with HRS type 1 and HRS type 2 to terlipressin or norepinephrine and the results to each condition were published in separated papers. Two studies [9], [10] classified the patients according to the first version of the International Ascites Club criteria [13] and the remaining [11], [12] by the updated criteria [14].

**Table 1:** Included studies.

Study	Design	Screened patients	Included patients	Terlipressin dosage	Norepinephrine dosage
Alessandria,2007 [9]	Single center, unblinded	36	20	1–2 mg every 4 h	0.05–0.7 mcg/kg/min
Sharma, 2008 [10]	Single center, unblinded	49	40	0.5–2 mg every 6 h	0.5–3 mg/h
Singh, 2012 [11]	Single center, unblinded	60	46	0.5–2 mg every 6 h	0.5–3 mg/h
Ghosh, 2013 [12]	Single center, unblinded	58	46	0.5–2 mg every 6 h	0.5–3 mg/h

In all studies, the norepinephrine infusion was adjusted to reach an increase of at least 10 mmHg in MAP. In three studies, norepinephrine infusion was also adjusted in order to reach a urine output of over 200 ml [10], [11], [12]. Norepinephrine infusion was increased every 4 h to reach these targets in all studies. Terlipressin was administered in fixed doses which could be increased every 3 days to decrease basal value of creatinine by at least 25% [9] or at least 1 mg/dl [10], [11], [12]. Norepinephrine and terlipressin were administered until the reversal of HRS or for a maximum of 15 days. In all studies, patients were administered intravenous albumin and had central venous pressure (CVP) measurements. Albumin was used to maintain a CVP of 10–15 cm H_2_O in the Italian study [9]. In the Indian studies, patients were given 20–40 g of albumin per day, which was discontinued if CVP was more than 18 cm H_2_O [10], [11], [12].

Table 2 shows the characteristics of the patients in each study.

**Table 2:** Characteristics of the included patients.

Study	Alessandria et al., 2007 [9]		Sharma et al., 2008 [10]		Singh et al., 2012 [11]		Ghosh et al., 2013 [12]	
	Norepinephrine(n = 10)	Terlipressin(n = 12)	Norepinephrine(n = 20)	Terlipressin(n = 20)	Norepinephrine(n = 23)	Terlipressin(n = 23)	Norepinephrine(n = 23)	Terlipressin(n = 23)
Age (years)	56±3	55±2	48.2±13.4	47.8±9.8	51.4±11.6	48.3±11.6	45.8±9.2	48.2±10.5
Etiology, Alcohol	2 (20.0%)	4 (33.3%)	12 (60.0%)	14 (70.0%)	10 (43.4%)	12 (52.1%)	15 (65.2%)	16 (69.6%)
Child Pugh score	10±1	11±1	11.0±0.9	10.6±0.8	10.70±2.01	10.43±1.72	10.0±1.77	10.5±2.35
MELD score	26±1	26±2	31.6±6.0	29.6±6.2	26.39±3.13	24.65±5.31	21.3±2.79	21.0±3.28
Serum creatinine (md/dl)	2.3±0.2	2.5±0.3	3.3±1.3	3.0±0.5	3.27±0.71	3.10±0.66	2.15±0.21	2.05±0.22
MAP (mmHg)	71±2	74±3	78.2±5.3	81.4±11.4	64.7±11.9	65.2±10.2	65.3±7.2	66.2±9.5

Data are mean ± standard deviation or number (%) of patients; MELD, model for end-stage liver disease; MAP, mean arterial pressure.

### Risk of bias

In table 3, the methodology of the quality assessment for each trial is reported using the Cochrane risk of bias tool. All studies were unblinded and eventually met the overall criteria for high risk of bias.

**Table 3:** Risk of bias assessment.

Study	Sequencegeneration	Allocationconcealment	Blinding of participants,personnel and outcomeassessors	Incomplete outcomedata	Selective outcomereporting	Other source ofbias	Overall risk ofbias
Alessandria, 2007 [9]	Unclear	Low	High	Low	Low	Unclear	High
Sharma, 2008 [10]	Low	Unclear	High	Low	Low	Unclear	High
Singh, 2012 [11]	Low	Low	High	Low	Low	Low	High
Ghosh, 2013 [12]	Low	Low	High	Low	Low	Low	High

### Outcomes

**Figure 2:**
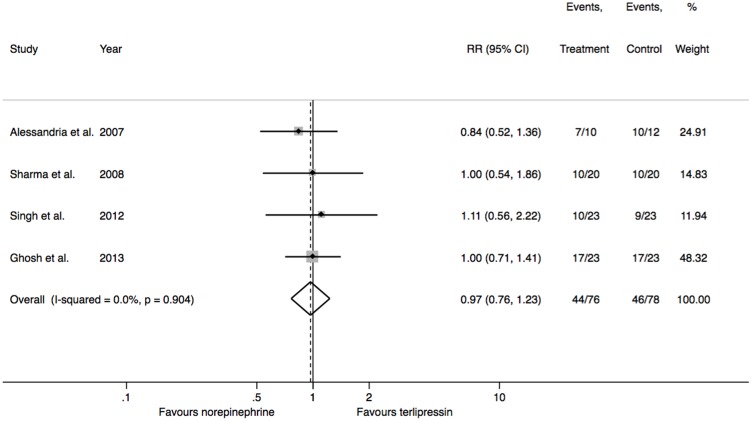
Reversal of hepatorenal syndrome. P values presented are for heterogeneity. P value for overall effect = 0.792. Chi-square = 0.536 (degrees of freedom = 3).

Reversal of HRS was assessed in 154 patients. There was no difference in the reversal of HRS between norepinephrine or terlipressin (RR = 0.97, 95% CI = 0.76 to 1.23; p = 0.800; I^2^ = 0%) (Figure 2). Ninety-five patients with type 1 HRS were included in three studies. There was also no difference in the reversal of HRS between norepinephrine and terlipressin in these patients (RR = 1.01, 95% CI = 0.69 to 1.49; p = 0.943; I^2^ = 0%). Fifty-nine patients with type 2 HRS were included in two trials and no difference between treatments could be demonstrated (RR = 0.95, 95% CI = 0.70 to 1.28; p = 0.717; I^2^ = 0%).

**Figure 3:**
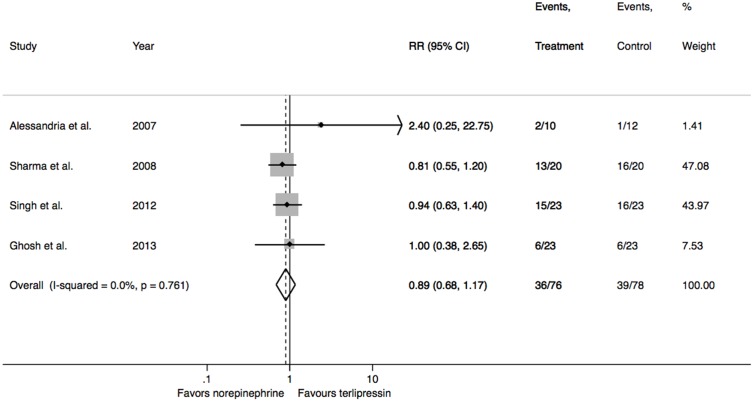
Mortality rates at 30 days. P values presented are for heterogeneity. P value for overall effect = 0.618. Chi-square = 1.077 (degrees of freedom = 3).

Since all studies reported the mortality rate at 30 days, this end-point was chosen to perform a pooled estimate. No difference in mortality at 30 days between norepinephrine and terlipressin could be found (RR = 0.89, 95% CI = 0.68 to 1.17; p = 0.404; I^2^ = 0%) (Figure 3). There were also no differences in mortality among subgroups of type 1 (RR = 0.88, 95% CI = 0.66 to 1.15; p = 0.345; I^2^ = 0%) and type 2 HRS patients (RR = 1.12, 95% CI = 0.44 to 2.83; p = 0.808; I^2^ = 0%).

Three studies reported recurrence rates of HRS after the cessation of the treatment [9], [11], [12]. There was no difference in these rates between norepinephrine and terlipressin (RR = 0,72; 95% CI = 0,36 to 1,15; p = 0.357; I^2^ = 0%) nor was among the subgroups of type 1 (RR = 0.71, 95% CI = 0.13 to 3.82; p = 0.688; I^2^ = 0%) and type 2 HRS patients (RR = 0.82, 95% CI = 0.036 to 1.84; p = 0.63; I^2^ = 0%).

**Figure 4:**
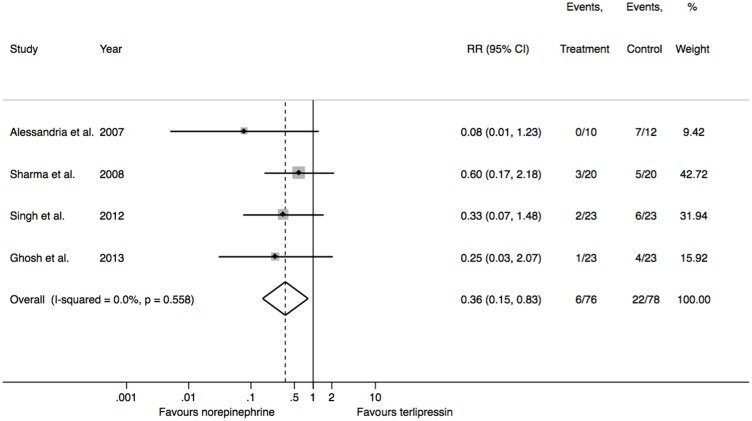
Adverse events. P values presented are for heterogeneity. P value for overall effect = 0.004. Chi-square = 1.901 (degrees of freedom = 3).

Adverse events were less common with norepinephrine (OR = 0.36, 95% CI 0.15 to 0.83; p = 0.017; I^2^ = 0%) (Figure 4), although all adverse events were of minor importance (Norepinephrine: three episodes of chest pain without electrocardiogram changes or troponin elevation, two episodes of ventricular extrasystoles, one episode of ST segment depression reversed after titration of the dose; terlipressin: 17 episodes of abdominal cramps and increased frequency of stools, two episodes of cyanosis, two episodes of extrasystoles and one episode of ST segment depression reversed after a titration of dose).

## Discussion

The results of this review suggest that in patients with HRS, treatment with norepinephrine is as effective as terlipressin when used in conjunction with albumin. Additionally, norepinephrine seems to be associated with less adverse events than terlipressin. However, these results are based on few trials with a reduced number of patients included.

In patients with cirrhosis, functional kidney failure is caused by a severe reduction of the effective circulating volume due to splanchnic arterial dilation and a reduction in the renal blood flow due to marked multifactorial intrarenal vasoconstriction [15]. This particular form of renal dysfunction develops in the later phases of liver failure and is characterized by low arterial pressure, intense activation of the renin-angiotensin and sympathetic nervous systems with an increase in the plasma levels of renin, norepinephrine, water retention due to increased anti-diuretic hormone and lowering glomerular filtration rates [1]. Without treatment, short-term mortality exceeds 50% with a median survival time of only 2 weeks [16].

Therapy with systemic vasoconstrictors and albumin is a bridging option to ameliorate renal dysfunction and to improve survival of patients while waiting for definitive treatment with liver transplantation. The rationale of associating these two therapies is to reduce the discrepancy between circulatory capacitance and intravascular volume, thereby increasing the effective arterial blood volume. Terlipressin promotes vasoconstriction in both systemic and splanchnic circulation through activation of V1 receptors of the vascular smooth muscle cells and is reported to reduce portal inflow, portal systemic shunting [17]; and to dilate intrahepatic vessels, consequently reducing intrahepatic resistance to portal inflow [18]. The overall results of the use of terlipressin in conjunction with albumin in the treatment of HRS are an improvement in renal function and an increase in the median survival time as demonstrated in clinical trials and confirmed by at least three meta-analyses [4], [19], [20]. Although terlipressin has become the vasoactive drug of choice where available, a Cochrane meta-analysis has pointed out that all randomized controlled studies that addressed the efficacy of terlipressin were underpowered and at high risk of bias [4]. Additionally, the evidence on the use of terlipressin in type 2 HRS is scarce since these patients were included in only one trial [21].

Norepinephrine, an inexpensive α-adrenergic receptor agonist available worldwide, is a possible alternative treatment for HRS because its intense vasoconstriction action may increase the effective arterial blood volume. A pilot single-center study with 12 patients demonstrated the reversal of HRS in 10 (83%) patients [5]. Since then, according to our literature search, four studies that aimed to compare norepinephrine and terlipressin in treatment of HRS have been published [9], [10], [11], [12].

Reversal of HRS occurred in 58% (Figure 2) of type 1 HRS patients treated with norepinephrine. These figures are very similar to the response rates reported on terlipressin arms of randomized controlled trials of this drug compared to placebo [4], but higher than those found in clinical practice [2], [3]. The trial of Ghosh et al. [12] was the first to randomize type 2 HRS patients exclusively. Response rates in this trial (74%) were higher than those found in type 1 HRS patients [12].Type 2 HRS patients included in the study published by Alessandria et al. also had a similar response (77%) to both vasoconstrictors [9].

Thirty day-mortality rates were around 50%. Two studies that included only type 1 HRS patients found a 30 day-mortality rate of over 65% [10], [11], which is similar to the ones reported in randomized controlled trials of terlipressin compared with a placebo [4], [19], but lower than clinical survey data [2], [3]. Recurrence rates were around 30%, similar to those found in observational studies [2], [22], but higher than those reported in the largest study which compared terlipressin and placebo [23].

Norepinephrine was associated with less adverse events than terlipressin. This difference was related to the frequency of abdominal cramps and diarrhea found in patients who were given terlipressin (17 cases in 78 patients). These are common adverse events related to terlipressin and are usually self-limiting, but were more common in our meta-analysis than in the Cochrane meta-analysis of terlipressin compared to placebo [4]. Norepinephrine and terlipressin both have a safe cardiovascular profile. Only nine cardiovascular events were found in the included trials and only two of them (episodes of segment ST depression) led to a change in therapy (a titration of dose) (10). Cardiovascular adverse effect rates were lower than those reported for terlipressin in the meta-analysis previously cited [4].

Although it was not among the outcomes of this review, we observed all included trials reported lower costs with norepinephrine than with terlipressin. However, all of them were performed in specialized units with a high level of surveillance and only costs related to the drugs were reported. Although more expensive, terlipressin has some advantages over norepinephrine. It is given as an intravenous bolus in a peripheral vein. This means that terlipressin can be safely used in regular wards. Norepinephrine is given intravenously as a continuous infusion in a central venous catheter, usually in the setting of intensive care unit. Therefore, a comparison of costs between these two treatments must also take into account intensive care costs.

In spite of an extensive literature search without language restriction that was conducted, we were not able to identify any studies published in non-indexed journals or as conference proceedings. Although included studies had no evidence of significant heterogeneity, and used similar treatment protocols, they had small sample sizes and were single-centered. Three of them were performed at a same center [10], [11], [12] and they included patients with different HRS criteria, as these were updated from 1996 to 2007 [13], [14]. Therefore, the first two studies adopted the first criteria [9], [10] and the remaining, the updated criteria [11], [12]. Undoubtedly, these findings reduce external validity of the results of this meta-analysis. Additionally, it would be questionable to combine data from patients with patients with type 1 and type 2 HRS since these two conditions have a different course and different responses to vasoconstrictors [1], [2], [3]. Similar limitations were also acknowledged in the meta-analyses of terlipressin compared to a placebo or other drugs in the treatment of HRS [4], [19]. In order to better address the question of efficacy and safety of terlipressin and norepinephrine in the treatment of type 1 and type 2 HRS, we have performed subgroup analysis on each condition.

Since the largest randomized study published with HRS patients included only 112 patients [23], a collaborative research network would be necessary to perform a large clinical trial comparing norepinephrine to terlipressin in the treatment of HRS.

In conclusion, norepinephrine and terlipressin had similar response rates for the treatment of type 1 or 2 HRS. However, norepinephrine was associated with less adverse events than terlipressin. Nevertheless, these findings are based on small studies, with a total of only 154 patients. A larger randomized controlled trial would be needed to draw firm conclusions on the choice of the vasoconstrictor to treat HRS.

## Supporting Information

Checklist S1 **PRISMA checklist.**
(DOC)
