# Supplementary material for: Terlipressin versus Norepinephrine in the Treatment of Hepatorenal Syndrome: A Systematic Review and Meta-Analysis
Source: PLoS One. 2014 Sep 9;9(9):e107466. doi: 10.1371/journal.pone.0107466 (PMC4159336; doi:10.1371/journal.pone.0107466)
Supplement: Checklist S1 — PRISMA checklist. (DOC) [file pone.0107466.s001.doc]

| **Section/topic** | **#** | **Checklist item** | **Reported on page #** |
| --- | --- | --- | --- |
| **TITLE** | | |  |
| Title | 1 | Terlipressin versus norepinephrine in the treatment of hepatorenal syndrome: a systematic review and meta-analysis | Title |
| **ABSTRACT** | | |  |
| Structured summary | 2 | **Background:** Hepatorenal syndrome (HRS) is a severe and progressive functional renal failure occurring in patients with cirrhosis and ascites. Terlipressin is recognized as an effective treatment of HRS, but it is expensive and not widely available. Norepinephrine could be an effective alternative. This systematic review and meta-analysis aimed to evaluate the efficacy and safety of norepinephrine compared to terlipressin in the management of HRS.  **Methods:** We searched the Medline, Embase, Scopus, CENTRAL, Lilacs and Scielo databases for randomized trials of norepinephrine and terlipressin in the treatment of HRS up to January 2014. Two reviewers collected data and assessed the outcomes and risk of bias. The primary outcome was the reversal of HRS. Secondary outcomes were mortality, recurrence of HRS and adverse events.  **Results:** Four studies comprising 154 patients were included. All trials were considered to be at overall high risk of bias. There was no difference in the reversal of HRS (RR = 0.97, 95% CI = 0.76 to 1.23), mortality at 30 days (RR = 0.89, 95% CI = 0.68 to 1.17) and recurrence of HRS (RR = 0.72; 95% CI = 0.36 to 1.45) between norepinephrine and terlipressin. Adverse events were less common with norepinephrine (RR = 0.36, 95% CI = 0.15 to 0.83).  **Conclusions**: Norepinephrine seems to be an attractive alternative to terlipressin in the treatment of HRS and is associated with less adverse events. However, these findings are based on data extracted from only four small studies.  **PROSPERO register**: CRD42013006723. | Abstract |
| **INTRODUCTION** | | |  |
| Rationale | 3 | “Since the arterial vasodilation seems to be a key mechanism in the pathogenesis of HRS, vasoconstrictors have been used as a bridging therapy leading up to the definitive treatment; liver transplantation. The vasopressin analog terlipressin is the most widely studied drug, especially in type 1 HRS. However, it is expensive and unavailable in many countries. Norepinephrine, a catecholamine with predominantly alpha-adrenergic activity, is widely available, inexpensive and has been used for the treatment of HRS type 1 since 2002.” | Introduction |
| Objectives | 4 | “With the ominous prognosis of HRS and the high cost associated with terlipressin in mind, we performed a systematic review and meta-analysis to evaluate the efficacy and safety of norepinephrine compared to terlipressin in the treatment of HRS.” | Introduction |
| **METHODS** | | |  |
| Protocol and registration | 5 | PROSPERO registration: CRD42013006723  Available online: http://www.crd.york.ac.uk/PROSPERO/display_record.asp?ID=CRD42013006723#.U_XbIsVdXQA | Methods |
| Eligibility criteria | 6 | “The search strategy was restricted to randomized clinical trials performed on adult subjects (…).” | Methods   - Literature search |
| Information sources | 7 | “Studies were identified through a search of the Medline, EMBASE, Scopus, Cochrane Central Register of Controlled Trials (CENTRAL), Lilacs (*Literatura Latino-Americana e do Caribe em Ciências da Saúde*) and Scielo (*Scientific Eletronic Library Online*) databases. (…) The search strategy was restricted to randomized clinical trials performed on adult subjects and published before 14 January 2014. There was no language restriction.”  “Authors of the included studies were contacted by email to complete the missing data that was required for characterizing the studies.” | Methods   - Literature search - Data extraction and quality assessment |
| Search | 8 | “A sensitive search strategy was used, combining the following Medical Subject Headings and keywords: “terlipressin” and “norepinephrine” or “noradrenalin” in combination with “hepatorenal syndrome”. References of the included studies were also searched” | Methods   - Literature search |
| Study selection | 9 | ““Studies that fulfilled the following criteria were included:   1. Compared terlipressin to norepinephrine in the treatment of type 1 or type 2 HRS; 2. Reported at least one of the following outcomes: reversal of HRS, effect on mortality, recurrence rates after cessation of the treatment or assessment of adverse events on both arms of the study.”” | Methods   - Study selection |
| Data collection process | 10 | “A data extraction sheet was developed. Two authors (APNJ and LMSM) independently extracted the following data from included studies, as available: year of publication, number of patients designated to terlipressin or norepinephrine, methods of randomization, allocation concealment, blinding method, age, type of HRS, etiology of cirrhosis and duration of treatment. Child-Pugh and MELD scores, serum creatinine and mean arterial pressure (MAP) were recorded at baseline. Authors of the included studies were contacted by email to complete the missing data that was required for characterizing the studies.” | Methods   - Data extraction and quality assessment |
| Data items | 11 | “The primary outcome was the reversal of HRS, defined as a decrease in the serum creatinine value to 133μmol/l (1.5 mg/dl) or lower during the treatment. Secondary outcomes were mortality, recurrence of HRS and adverse effects. “ | Methods   - Outcomes |
| Risk of bias in individual studies | 12 | “Two authors (APNJ and LMSM) assessed the risk of bias of individual trials using the Cochrane risk of bias tool. For the outcomes in each included trial, the risk of bias was reported as ‘low risk’, ‘unclear risk’, or ‘high risk’ in the following domains: random sequence generation; allocation concealment; blinding of participants and personnel; blinding of outcome assessment; incomplete outcome data; selective reporting; or other bias” | Methods Data extraction and quality assessment |
| Summary measures | 13 | “Differences observed between the treatment groups were expressed as the pooled risk ratio (RR) with a 95% confidence interval (CI).” | Methods   - Statistical analysis |
| Synthesis of results | 14 | “Heterogeneity was assessed by the I^2^ statistic. A random-effects model was employed due to the anticipated variability between trials in terms of patient populations, interventions, and concomitant interventions. The effect of the treatment on the defined outcome measures was calculated from the raw data using random effects models.” | Methods   - Statistical analysis |

Page 1 of 2

| **Section/topic** | **#** | **Checklist item** | **Reported on page #** |
| --- | --- | --- | --- |
| Risk of bias across studies | 15 | NA | - |
| Additional analyses | 16 | “*A priori* subgroup analysis was performed to assess reversal, mortality and recurrence of type 1 and type 2 HRS.” | Methods   - Statistical analysis |
| **RESULTS** | | |  |
| Study selection | 17 | “The search yielded 77 publications. Four randomized controlled trials were selected for the analysis (Figure 1)” | Results (Trial identification) and Figure 1 |
| Study characteristics | 18 | “Table 1 summarizes the details of included studies. One study was performed in Italy and the remaining three were performed at the same center in India. Two studies included patients with type 1 HRS , one with type 2 HRS and one with both types of HRS . The studies performed by Singh et al. and Ghosh et al. were actually a single center trial which randomized patients with HRS type 1 and HRS type 2 to terlipressin or norepinephrine and the results to each condition were published in separated papers. Two studies classified the patients according to the first version of the International Ascites Club criteria and the remaining by the updated criteria.  In all studies, the norepinephrine infusion was adjusted to reach an increase of at least 10mmHg in MAP. In three studies, norepinephrine infusion was also adjusted in order to reach a urine output of over 200ml . Norepinephrine infusion was increased every 4h to reach these targets in all studies. Terlipressin was administered in fixed doses which could be increased every 3 days to decrease basal value of creatinine by at least 25% or at least 1mg/dl . Norepinephrine and terlipressin were administered until the reversal of HRS or for a maximum of 15 days. In all studies, patients were administered intravenous albumin and had central venous pressure (CVP) measurements. Albumin was used to maintain a CVP of 10-15cmH2O in the Italian study . In the Indian studies, patients were given 20-40g of albumin per day, which was discontinued if CVP was more than 18cmH2O .  Table 2 shows the characteristics of the patients in each study.” | Results (Trial characteristics) and tables 1,2 |
| Risk of bias within studies | 19 | “In table 3, the methodology of the quality assessment for each trial is reported using the Cochrane risk of bias tool. All studies were unblinded and eventually met the overall criteria for high risk of bias.” | Results (Risk of bias) and table 3 |
| Results of individual studies | 20 | Figures 2,3 and 4 | Figures 2, 3 and 4 |
| Synthesis of results | 21 | “Reversal of HRS was assessed in 154 patients. There was no difference in the reversal of HRS between norepinephrine or terlipressin (RR = 0.97, 95% CI = 0.76 to 1.23; p = 0.800; I^2^ = 0%)(Figure 2).  Since all studies reported the mortality rate at 30 days, this end-point was chosen to perform a pooled estimate. No difference in mortality at 30 days between norepinephrine and terlipressin could be found (RR = 0.89, 95% CI = 0.68 to 1.17; p = 0.404; I^2^ = 0%) (Figure 3).  Three studies reported recurrence rates of HRS after the cessation of the treatment. There was no difference in these rates between norepinephrine and terlipressin (RR = 0,72; 95% CI = 0,36 to 1,15; p = 0.357; I^2^ = 0%) .  Adverse events were less common with norepinephrine (OR = 0.36, 95% CI 0.15 to 0.83; p = 0.017; I2 = 0%)(Figure 4), although all adverse events were of minor importance (Norepinephrine: three episodes of chest pain without electrocardiogram changes or troponin elevation, two episodes of ventricular extrasystoles, one episode of ST segment depression reversed after titration of the dose; terlipressin: 17 episodes of abdominal cramps and increased frequency of stools, two episodes of cyanosis, two episodes of extrasystoles and one episode of ST segment depression reversed after a titration of dose).” | Results (Outcomes), Figures 2-4 |
| Risk of bias across studies | 22 | NA | - |
| Additional analysis | 23 | “Ninety-five patients with type 1 HRS were included in three studies. There was also no difference in the reversal of HRS between norepinephrine and terlipressin in these patients (RR = 1.01, 95% CI = 0.69 to 1.49; p = 0.943; I^2^ = 0%). Fifty-nine patients with type 2 HRS were included in two trials and no difference between treatments could be demonstrated (RR = 0.95, 95% CI = 0.70 to 1.28; p = 0.717; I^2^ = 0%).  There were also no differences in mortality among subgroups of type 1 (RR = 0.88, 95% CI = 0.66 to 1.15; p = 0.345; I^2^ = 0%) and type 2 HRS patients (RR = 1.12, 95% CI = 0.44 to 2.83; p = 0.808; I^2^ = 0%).  Three studies reported recurrence rates of HRS after the cessation of the treatment. There was no difference in these rates between norepinephrine and terlipressin among the subgroups of type 1 (RR = 0.71, 95% CI = 0.13 to 3.82; p = 0.688; I^2^ = 0%) and type 2 HRS patients (RR = 0.82, 95% CI = 0.036 to 1.84; p = 0.63; I^2^ = 0%).” | Results (Outcomes) |
| **DISCUSSION** | | |  |
| Summary of evidence | 24 | “The results of this review suggest that in patients with HRS, treatment with norepinephrine is as effective as terlipressin when used in conjunction with albumin. Additionally, norepinephrine seems to be associated with less adverse events than terlipressin. However, these results are based on few trials with a reduced number of patients included.” | Discussion |
| Limitations | 25 | “In spite of an extensive literature search without language restriction that was conducted, we were not able to identify any studies published in non-indexed journals or as conference proceedings. Although included studies had no evidence of significant heterogeneity, and used similar treatment protocols, they had small sample sizes and were single-centered. Three of them were performed at a same center and they included patients with different HRS criteria, as these were updated from 1996 to 2007. Therefore, the first two studies adopted the first criteria and the remaining, the updated criteria. Undoubtedly, these findings reduce external validity of the results of this meta-analysis. Additionally, it would be questionable to combine data from patients with patients with type 1 and type 2 HRS since these two conditions have a different course and different responses to vasoconstrictors.” | Discussion |
| Conclusions | 26 | “In conclusion, norepinephrine and terlipressin had similar response rates for the treatment of type 1 or 2 HRS. However, norepinephrine was associated with less adverse events than terlipressin. Nevertheless, these findings are based on small studies, with a total of only 154 patients. A larger randomized controlled trial would be needed to draw firm conclusions on the choice of the vasoconstrictor to treat HRS.” | Discussion |
| **FUNDING** | | |  |
| Funding | 27 | There was no funding for this study | - |
